# De novo transcriptome characterization of *Iris atropurpurea* (the Royal Iris) and phylogenetic analysis of MADS-box and R2R3-MYB gene families

**DOI:** 10.1038/s41598-021-95085-5

**Published:** 2021-08-10

**Authors:** Yamit Bar-Lev, Esther Senden, Metsada Pasmanik-Chor, Yuval Sapir

**Affiliations:** 1 The Botanical Garden, School of Plant Sciences and Food Security, G.S. Wise Faculty of Life Science, Tel Aviv University, Tel Aviv, Israel; 2 Bioinformatics Unit, G.S. Wise Faculty of Life Science, Tel Aviv University, 69978 Tel Aviv, Israel

**Keywords:** Transcriptomics, Molecular evolution

## Abstract

The Royal Irises (section *Oncocyclus)* are a Middle-Eastern group of irises, characterized by extremely large flowers with a huge range of flower colors and a unique pollination system. The Royal Irises are considered to be in the course of speciation and serve as a model for evolutionary processes of speciation and pollination ecology. However, no transcriptomic and genomic data are available for these plants. Transcriptome sequencing is a valuable resource for determining the genetic basis of ecological-meaningful traits, especially in non-model organisms. Here we describe the de novo transcriptome assembly of *Iris atropurpurea*, an endangered species endemic to Israel’s coastal plain. We sequenced and analyzed the transcriptomes of roots, leaves, and three stages of developing flower buds*.* To identify genes involved in developmental processes we generated phylogenetic gene trees for two major gene families, the MADS-box and MYB transcription factors, which play an important role in plant development. In addition, we identified 1503 short sequence repeats that can be developed for molecular markers for population genetics in irises. This first reported transcriptome for the Royal Irises, and the data generated, provide a valuable resource for this non-model plant that will facilitate gene discovery, functional genomic studies, and development of molecular markers in irises, to complete the intensive eco-evolutionary studies of this group.

## Introduction

*Iris* is the largest genus in the Iridaceae (Asparagales) with over 300 species^1,2^. The genus is highly heterogeneous, with species exhibiting a wide range of plant sizes, and flower shapes and colors^1^.

The Royal Irises (*Iris* section *Oncocyclus*) are a Middle-Eastern group of about 32 species that are endemics to dry, Mediterranean-type climates and found in the eastern Mediterranean Basin, Caucasica, and central Anatolia^3^. Species of section *Oncocyclus* in Israel occur in small isolated populations and many are considered rare, threatened, or endangered^4^. These species are characterized by a single large flower on a stem and perennial, short, knobby rhizomes, occasionally with stolons^3,5^. Plants are diploid with chromosome number of 2n = 20^6^. This number is relatively low for *Iris* species, whose chromosome number ranges from 2n = 16 in *I. attica* to 2n = 108 in *I. versicolor* (data obtained from Chromosome Count DataBase^7^)*,* and genome size ranges from 2,000 to 30,000 Mbp^8^.

The Royal Irises are thought to be undergoing recent speciation^3,5,9^. Consequently, in recent years, they have emerged as a platform for the study of evolutionary processes of speciation, adaptation and pollination ecology^3,10–17^. Evolutionary processes and adaptive phenotypes are governed by genetic differences. Thus, the study of plant ecology and evolution increasingly depends on molecular approaches, from identifying the genes underlying adaptation, reproductive isolation, and speciation, to population genetics. No genetic and molecular tools are yet available for the Royal Irises. Whole-genome sequencing of the *Iris* is a challenging task, due to its large genome size^8^, and therefore transcriptome sequencing may provide a feasible, still a strong genomic resource.

Transcriptome sequencing is a powerful tool for high-throughput gene discovery, and for uncovering the molecular basis of biological functions, in non-model organisms^18^. Few *Iris* transcriptomes have been already sequenced^19–22^*,* all are of irises which are in distant clades from *Oncocyclus* iris^23^. Currently, only one NGS-based dataset is available for the Royal Irises, which is a plastid genome sequence of *Iris gatesii*^24^. Previous attempts to transfer molecular tools developed for Louisiana irises to *Oncocyclus* irises, such as the development of microsatellite loci or identifying candidate genes, have failed (Y. Sapir, un-published). Furthermore, Royal Iris species have low plastid variance (Y. Sapir and Y. Bar-Lev, un-published) and lack nuclear sequences. All these, call for a wider set of molecular tools. Our main objective was to generate a reference RNA sequence for the Royal Irises that can serve as a molecular toolbox.

Here we report the de novo assembly of a transcriptome for *Iris atropurpurea* Baker, one of the Royal Irises species. *I. atropurpurea* is a highly endangered plant endemic to Israeli coastal plain^25,26^. In recent years this species has been studied extensively for its morphology^5^, pollination^14,15,27^, speciation, and population divergence^13,17^. In order to answer any further questions in this system, molecular tools are needed. Transcriptome sequencing of *I. atropurpurea* will facilitate further studies of genetic rescue, population genetics, as well as finding genes that underlie different biological functions.

One of the most important biological functions to understand plant evolution is plant development. To identify genes involved in developmental processes, we analyzed the phylogeny of sequences annotated to MADS-box and R2R3-MYB transcription factors families, which are involved in the regulation of diverse developmental functions. Homologs for genes of these families have been identified in *Iris fulva* of the Louisiana irises^19^. We therefore aimed to identify their homologs in the Royal Irises.

Plant development greatly depends on the function of MADS-box transcription factors, a very ancient family of DNA binding proteins, which are present in nearly all major eukaryotic groups. MADS-box genes comprise a highly conserved sequence of ~ 180 bp, which encodes the DNA binding domain in the MADS-box protein^28,29^. MADS-box genes are divided into type I and type II. In plants, type I MADS-box genes are subdivided into three groups: Mα, Mβ and Mγ^30,31^. They are involved in female gametophyte, embryo sac, and seed development. The type II MADS-box genes in plants are known as the MIKC MADS-box group and are extensively studied. MIKC proteins convey three additional distinctive regions: an intervening region (I), a keratin-like domain (K), and a C-terminal domain (C)^32,33^. Found within this group are the MIKCc and MIKC* subgroups^34^. MIKCc MADS-box genes (the c stands for classic), are mainly involved in plant and flower development^35,36^, and are phylogenetically divided into 14 major groups in *Arabidopsis* and rice^37,38^. The MIKC* group, in some reports, matches the *Arabidopsis* Mδ subgroup, defined as part of the type I group^39^.

Another superfamily of transcription factors, that are important for plant development, are the MYB proteins, which contain the conserved MYB DNA-binding domain^40^. The MYB family members are categorized based on the number of MYB domain repeats: 1R- (MYB related genes, containing a single or partial MYB domain), R2R3-, 3R- and 4R-MYB proteins^40–42^. MYB proteins are widely distributed in plants, in which the R2R3-MYB subfamily is the most abundant (containing an R2 and R3 MYB domain)^40,41,43^. The large abundance of the R2R3-MYB family in plants indicates their importance in the control of various plant specific processes, such as responses to biotic and abiotic stresses, development, defense reactions, flavonoid and anthocyanin biosynthesis, regulation of meristem formation, and floral and seed development (reviewed in^43^ and ^44^).

Here we employed phylogenetic approach to identify homologs of MADS-box and R2R3-MYB transcription factors in the *I. atropurpurea* transcripts. We sequenced transcriptomes from various tissues and flower bud developmental stages. From these, we established an annotated database for *I. atropurpurea*, potentially applicable to other species of the Royal Irises, and explored the homologs of MADS-box and R2R3-MYB. This is the first reported transcriptomes for the *Oncocyclus* section. The sequenced *Iris* transcriptome offers a new foundation for genetic studies and enables exploring new research questions.

## Materials and methods

### Plant material

**Figure 1:**
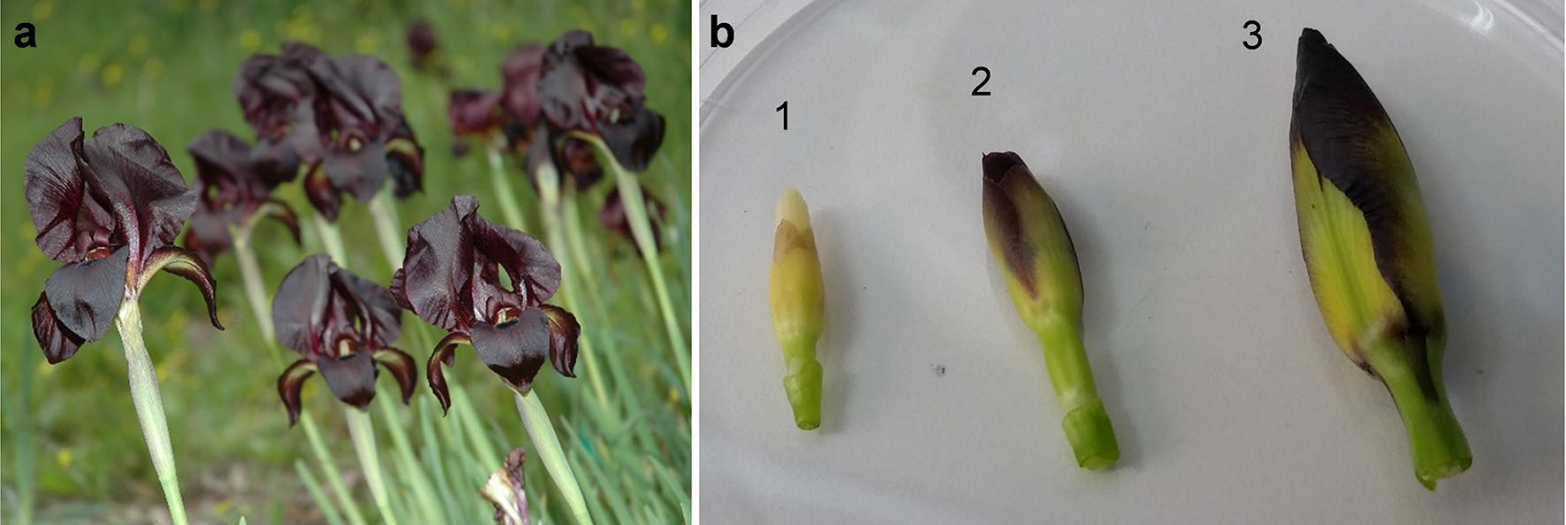
Plant materials used for RNA sequencing. (**a**) *Iris atropurpurea* flower in the field site where collected (Dora). (**b**) Representation of three stages of bud development (1 to 3) in *I. atropurpurea*, as defined in the text.

We used two accessions (genotypes) of *I. atropurpurea*, DR14 and DR8. Plants were brought from a large *I. atropurpurea* population in Dora (32° 17′ N 34° 50′ E) in Israel (Fig. 1a) and grown at the Tel Aviv University Botanical Garden. Aiming at finding genes related to flower development and floral traits, we used three different bud developmental stages. We defined bud developmental stage 1 as the earliest detectable bud, where the bud has no color, and is 1 cm in size. Stage 2 is a bud around 1.5 cm in size with the anthers still prominently visible above the petals, and at the onset of color production. Stage 3 is a full-colored bud, over 2 cm in size and with the petals covering the anthers (Fig. 1b). Earlier stages of flower development in the Royal Irises are nearly impossible to detect in naturally-growing plants. In these stages the meristem is attached to the rhizome underground and requires much destruction of the plant to be found^45^. We collected tissues from the root, young leaf and four buds in three developmental stages (one bud from stages 1 and 3, and two buds of stage 2) from DR14. We also collected buds in stages 1 and 2 from DR8 to enlarge the representation of rare or low expressed genes. Unfortunately, due to the low number of flowers (buds) per plant in Royal Irises, we were unable to obtain replicates for all bud stages. The collection of plant material complies with institutional guidelines and is coordinated with the Israel Nature and Parks Authority. *I. atropurpurea* lack voucher specimen, however, live plants are kept at Tel Aviv University Botanical Garden.

### RNA isolation and sequencing

We extracted total RNA from all the tissue samples using the RNeasy Mini Kit (Qiagen, Hilden, Germany), according to the manufacturer's instructions. We measured the quantity and quality of each RNA sample using Qubit fluorometer (Invitrogen) and Bioanalyzer TapeStation 2200 (Agilent Technologies Inc., USA), respectively. Only RNA samples that presented sufficient 260/280 and 260/230 purity and RIN (RNA integrity number) above 8.0 were used for sequencing. RNA was processed by the Technion Genome Center as following: RNA libraries were prepared using TruSeq RNA Library Prep Kit v2 (Illumina), according to manufacturer’s instructions, and libraries were sequenced using HiSeq 2500 (Illumina) on one lane of 100 PE run, using HiSeq V4 reagents (Illumina). Sequences generated in this study were deposited in NCBI’s Gene Expression Omnibus (GEO, http://www.ncbi.nlm.nih.gov/geo/) under the GEO accession number GSE121786.

### De novo transcriptome assembly and annotation

The quality of the raw sequence reads was estimated using FastQC (v 0.11.5, https://www.bioinformatics.babraham.ac.uk/projects/fastqc/). De novo assembly of the *Iris* transcriptome was done using Trinity (version trinityrnaseq_r20140717, https://github.com/trinityrnaseq/trinityrnaseq/wiki), with a minimum contig length of 200 base pairs (bp)^46^. We estimated assembly quality and completeness using Quast (v.3.2)^47^ and Benchmarking Universal Single-Copy Orthologs (BUSCO) (v 5.1.2, https://busco.ezlab.org/)^48^. Contigs (isoforms) that are likely to be derived from alternative splice forms or closely-related paralogs were clustered together by Trinity and referred to as “transcripts”. The initial reads from each sample were mapped back to the *Iris* transcriptome that was assembled, using trinity pipeline and Bowtie (v. 1.0.0, http://bowtie-bio.sourceforge.net/index.shtml). The number of mapped reads per transcript per sample was counted using RSEM (v. 1.2.25, http://deweylab.github.io/RSEM/)^49^.

To find the putative genes and function, transcripts were aligned against the UniProt non-redundant protein database (2016–09-26) and against PFAM protein family database^50^, using BLASTX alignment with an e-value cutoff to < 0.0001^51^. To classify functions of the transcripts, they were also aligned against the Gene Ontology (GO, http://geneontology.org/) and the Clusters of Orthologous Groups (COGs, https://www.ncbi.nlm.nih.gov/research/cog-project) protein databases. Annotations were computed using eggnog-mapper^52^, based on eggNOG 4.5 orthology data^53^. For transcription factors prediction, we submitted the sequences to search against PlantTFDB^54^.

### Phylogeny analysis

We retrieved all *Iris* transcripts that were annotated as either MADS or MYB proteins from the transcriptome and translated the longest open reading frame (ORF) using Virtual Ribosome^55^. We took the transcriptome transcripts that also contain the MADS or R2R3-MYB domain by PFAM. We downloaded *I. fulva* protein sequences for MIKCc MADS-box and R2R3-MYB transcription factors from NCBI^19^. *Arabidopsis* and rice (*oryza sativa*) MADS and R2R3-MYB sequences were taken from their genome databases [The *Arabidopsis* Information Resource (TAIR): www.arabidopsis.org and the Rice Genome Annotation Project (RGAP): rice.plantbiology.msu.edu, respectively]. The gene identifiers were denoted to AtMYB genes in *Arabidopsis* and the locus id in rice to avoid confusion when multiple names are used for same gene. The sequences of each gene family were trimmed using trimAl (v1.3, http://trimal.cgenomics.org/)^56^ and aligned using ClustalW alignment^57^, in MEGA X Molecular Evolutionary Genetics Analysis Software (https://www.megasoftware.net/)^58^. We tested for the best substitution model and found that the best model for MADS is the JTT (Jones, Taylor, Thornton) model^59^ + Gamma-distributed rates (G), and for MYB, JTT + G + amino acid frequency (F). For comparative phylogenetic analysis, we used maximum likelihood in MEGA X^58^ with 1000 bootstrap replications. Phylogenetic trees were visualized using FigTree (v1.4.3, http://tree.bio.ed.ac.uk/software/figtree/)^60^.

### SSRs mining

In order to utilize the transcriptome sequenced also for population genetic markers, we searched for short sequence repeats (SSRs; microsatellites) in the assembled contigs. We used a Perl script (find_ssrs.pl^61^) to identify microsatellites in the unigenes. In this study, SSRs were considered to contain motifs with two to six nucleotides in size and a minimum of four contiguous repeat units.

## Results and discussion

### Sequencing of *Iris* transcriptome

To generate the *Iris* transcriptome, eight cDNA libraries were sequenced: root, leaf and three bud stages from one genotype of *I. atropurpurea* (DR14), and buds in stages 1 and 2 from a different genotype of the same population (DR8). We generated a total of 195,412,179 sequence reads. The average GC content of *Iris* contigs was 47% (Tables 1 and 3). Reads were of very high quality throughout their length, without evidence of adapter content (Phred score > 30).

**Table 1:** Statistical summary of *Iris* transcriptome sequencing and assembly.

Total reads	195,412,179
Contigs (Isoforms)	258,466
Transcripts	184,341
Transcriptome size	168,049,166
N50 contig size (≥ 500 bp)	1,312
Largest contig	27,971

Using Trinity, we assembled 258,466 contigs (isoforms) longer than 200 bp, which clustered into 184,341 transcripts, with a total length of 168,049,166 bp. A larger N50 length and average length are considered indicative of better assembly. The longest contig was 27,971 bp and half of the contigs (N50) with more than 500 bp were above 1,312 bp long (Table 1).

**Figure 2:**
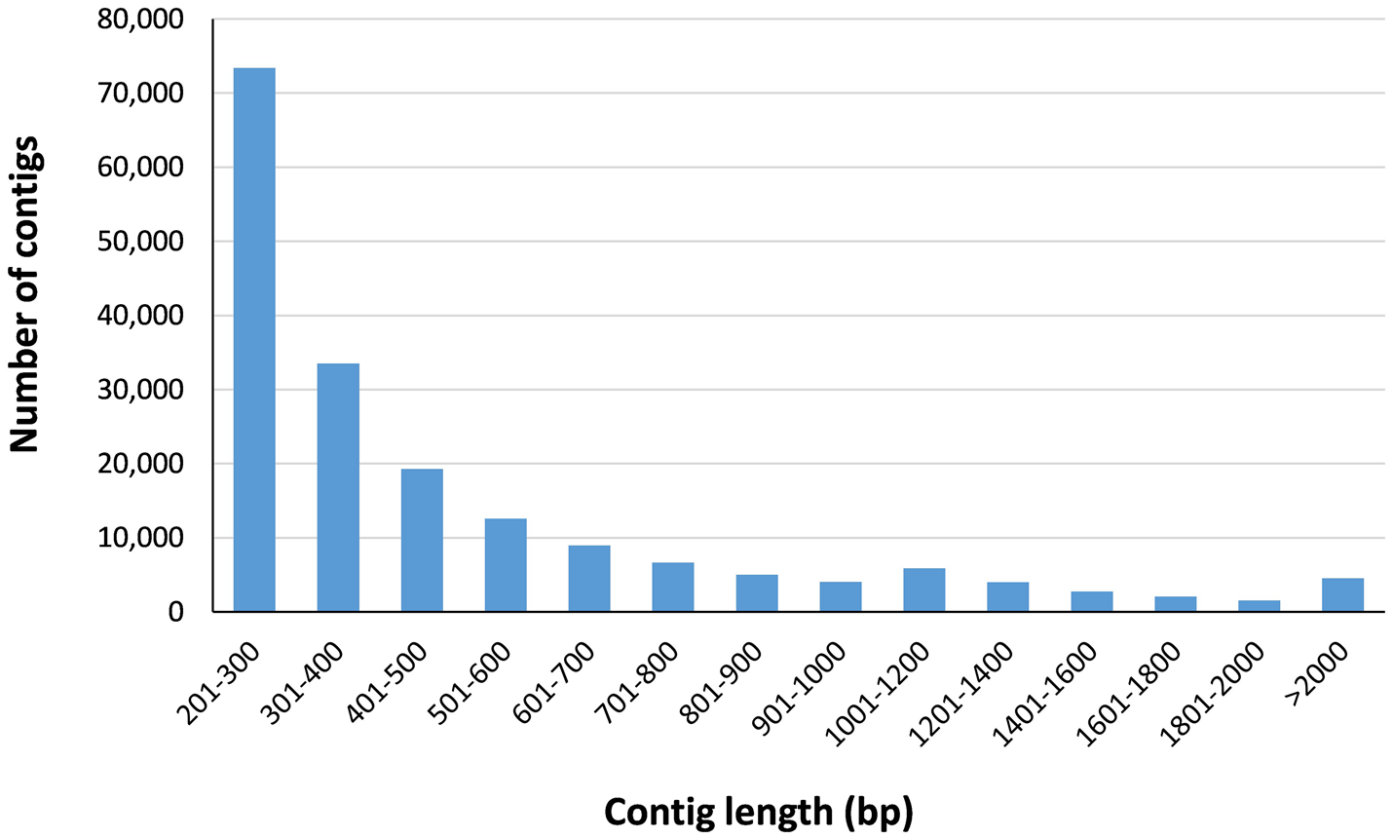
Distribution of contig lengths (in base pairs) across the assembled contigs from the *Iris* transcriptome.

The length distribution of the assembled contigs revealed that 126,194 (68.46%) contigs ranged from 201 to 500 bp in length; 37,335 (20.25%) contigs ranged from 501 to 1000 bp in length; 16,282 (8.83%) contigs ranged from 1001 to 2000 bp in length; and 4530 (2.46%) contigs were more than 2000 bp in length (Fig. 2). Subjecting our transcriptome to BUSCO analysis^48^ confirmed that our transcriptome assembly contains 82.4% of gene representation of the available orthologue groups at Liliopsida, and more than 90% at Embryophyta. Only 3 to 6.1% of the single-copy orthologs were classified as missing from our assembly, indicating high quality of the assembly (Table 2).

**Table 2:** BUSCO analysis of transcriptome completeness.

BUSCO (%)	Embryophyta	Liliopsida
Complete BUSCOs	90.4	82.4
Complete single copy	64.7	45.2
Duplicated	25.7	37.2
Fragmented	6.6	11.5
Missing	3	6.1

To quantify the abundance of contigs assembled, the reads of the separated *Iris* organs were mapped to the assembled contigs, with 125,074,925 (65%) mapped reads overall, and an average of 45% reads per tissue that mapped to a unique sequence in the assembled transcriptome (Table 3). Low mapping rates could be due to reads belonging to sequences below the 200 bp cut-off and also, presumably, due to the complexity of the *Oncocyclus* irises genome that is very large and highly repetitive^62,63^.

**Table 3:** Descriptive statistics of *Iris* transcriptome samples. GC—Percentage of G or C nucleotides in the sequence.

Plant ID	Tissue	# Paired-end sequences	#Reads	%GC	Total mapped reads	% Unique mapped reads
**DR14**	Root	47,760,556	23,880,278	48	16,671,086	55
	Leaf	52,936,482	26,468,241	47	18,366,659	54
	Bud stage 1	54,806,560	27,403,280	47	16,610,741	40
	Bud stage 2 (a)	51,092,390	25,546,195	47	16,095,948	43
	Bud stage 2 (b)	48,073,466	24,036,733	47	15,363,667	43
	Bud stage 3	59,105,996	29,552,998	47	18,570,119	43
**DR8**	Bud stage 1	36,949,342	18,474,671	45	10,887,290	40
	Bud stage 2	40,099,566	20,049,783	46	12,509,415	45

### Annotation of *Iris* transcriptome

**Figure 3:**
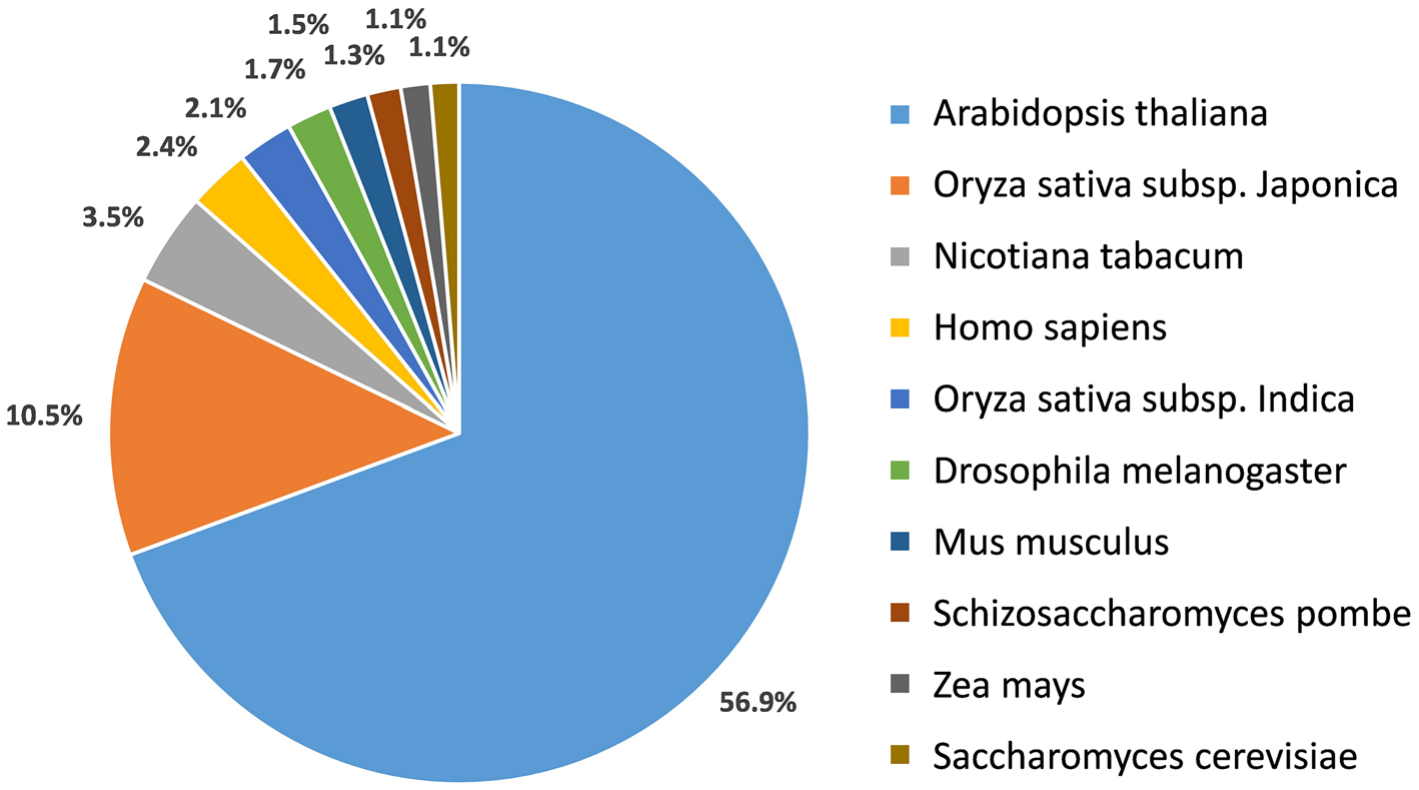
Top 10-hit species distribution of annotated transcripts. Other species represented in the transcriptome had only 1% or less of the transcripts annotated to them.

Using BLASTX search against the UniProt database, we identified 28,708 transcripts with at least one significant hit. Transcripts mostly annotated to *Arabidopsis thaliana* (56.9%)*, Oryza sativa* Japonica Group (10.5%) and *Nicotiana tabacum* (3.5%) (Fig. 3). Surprisingly, a significant proportion of the annotated transcripts were annotated as *Arabidopsis thaliana*, while only 10% were annotated as *Oryza sativa*, which is a monocot and therefore more closely related to irises. This is probably due to the higher representation of genomic resources for *Arabidopsis thaliana*. A considerable number of transcripts annotated to “non-plant” organisms, most of them to human (*Homo sapiens*, 2.4%) (Fig. 3). This may be attributed to housekeeping genes, which are preserved across all species in eukaryotes, and may also be due to the highly annotated human genome.

**Figure 4:**
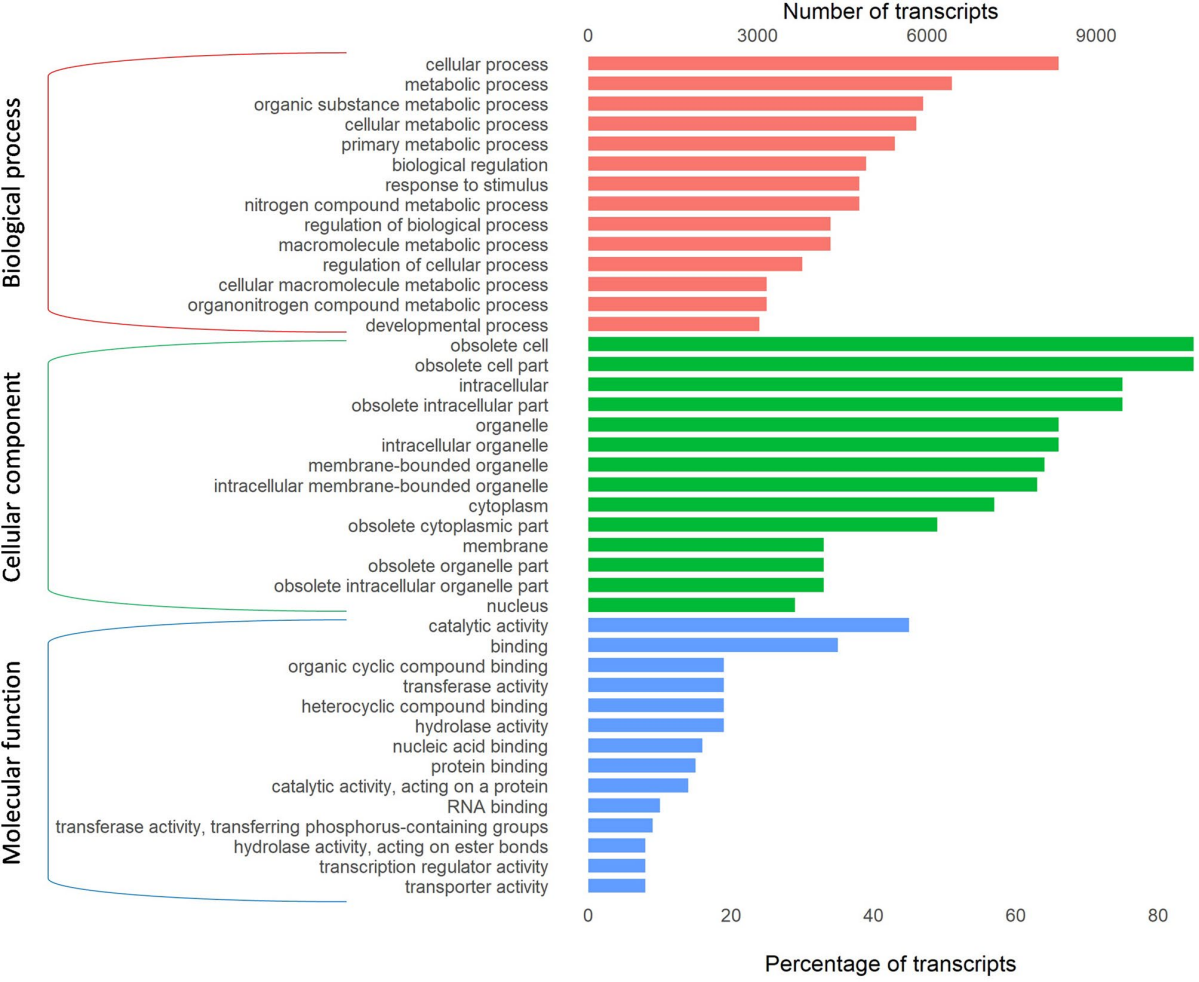
Clusters of orthologous group (COG) classification, showing 22,564 transcripts that were classified.

In the gene ontology analysis, 12,623 transcripts were assigned to GO terms under the three categories (supplementary table 1). Within the biological process category, ‘cellular process’ and ‘metabolic process’ were the two GO terms with the highest numbers of transcripts. In the cellular component category, ‘obsolete cell’ and ‘obsolete cell part’ were the most abundant. For the molecular function category, ‘catalytic activity’ and ‘binding’ had the highest number of transcripts (Fig. 4, supplementary info file).

**Figure 5:**
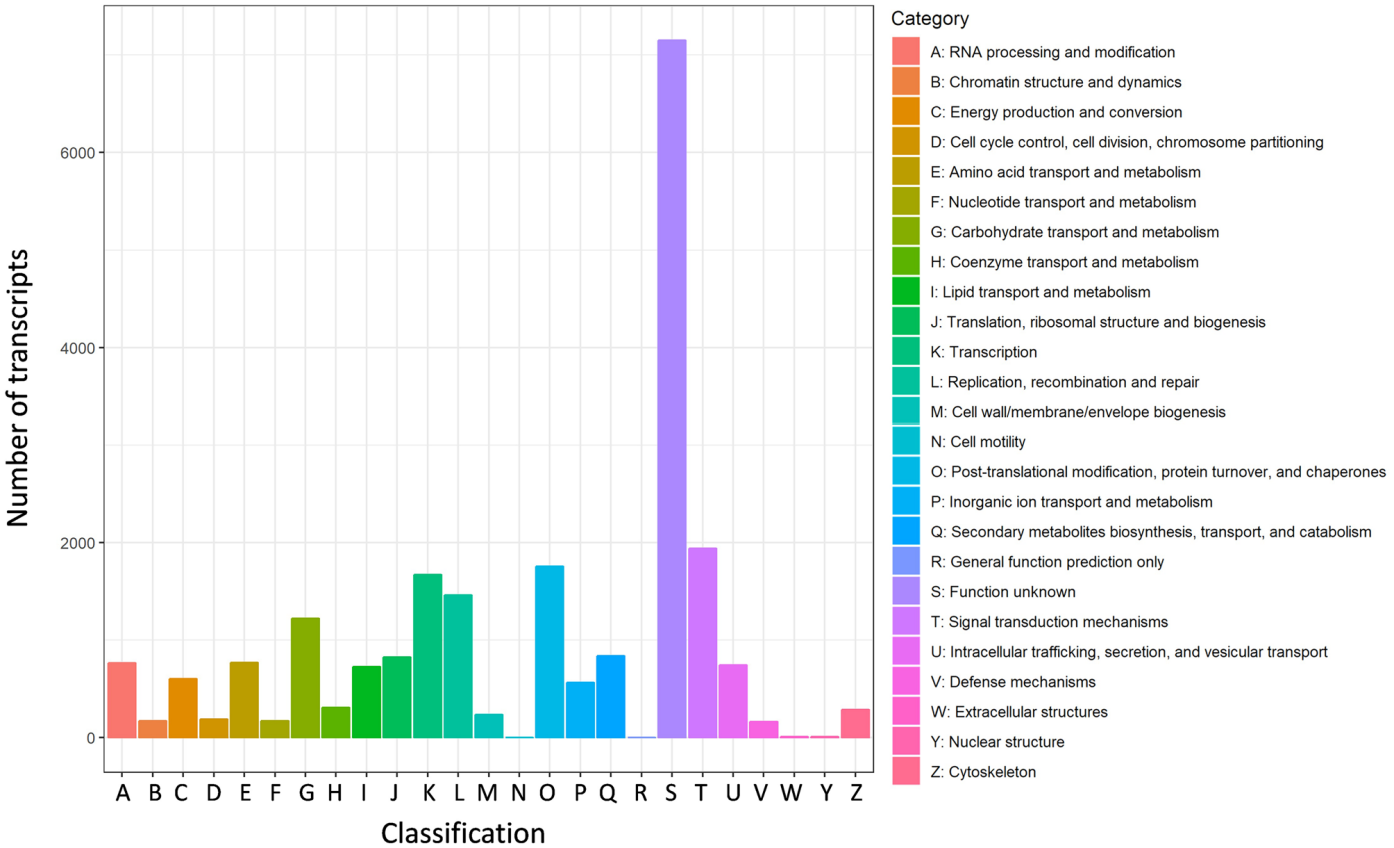
Clusters of orthologous group (COG) classification, showing 22,564 transcripts that were classified.

Search against the COG database resulted in the classification of 22,564 transcripts (supplementary table 1). Among the 25 COG categories, the cluster for unknown function was the largest group (7,151, 31.69%). The following categories of the top ten are: signal transduction mechanisms (1938, 8.59%), posttranslational modification, protein turnover and chaperones (1754, 7.77%), transcription (1670, 7.4%), replication, recombination and repair (1461, 6.47%), carbohydrate transport and metabolism (1222, 5.42%), secondary metabolites biosynthesis, transport and catabolism (839, 3.72%), translation, ribosomal structure and biogenesis (825, 3.66%), amino acid transport and metabolism (767, 3.4%), and RNA processing and modification (764, 3.39%) (Fig. 5, supplementary info file). The COG term ‘signal transduction’ was also enriched in previous transcriptomes, such as in *Iris lactea*^22^, *Camelina sativa* L^64^, and in *Taxodium* ‘Zhongshanshan 405′^65^.

**Figure 6:**
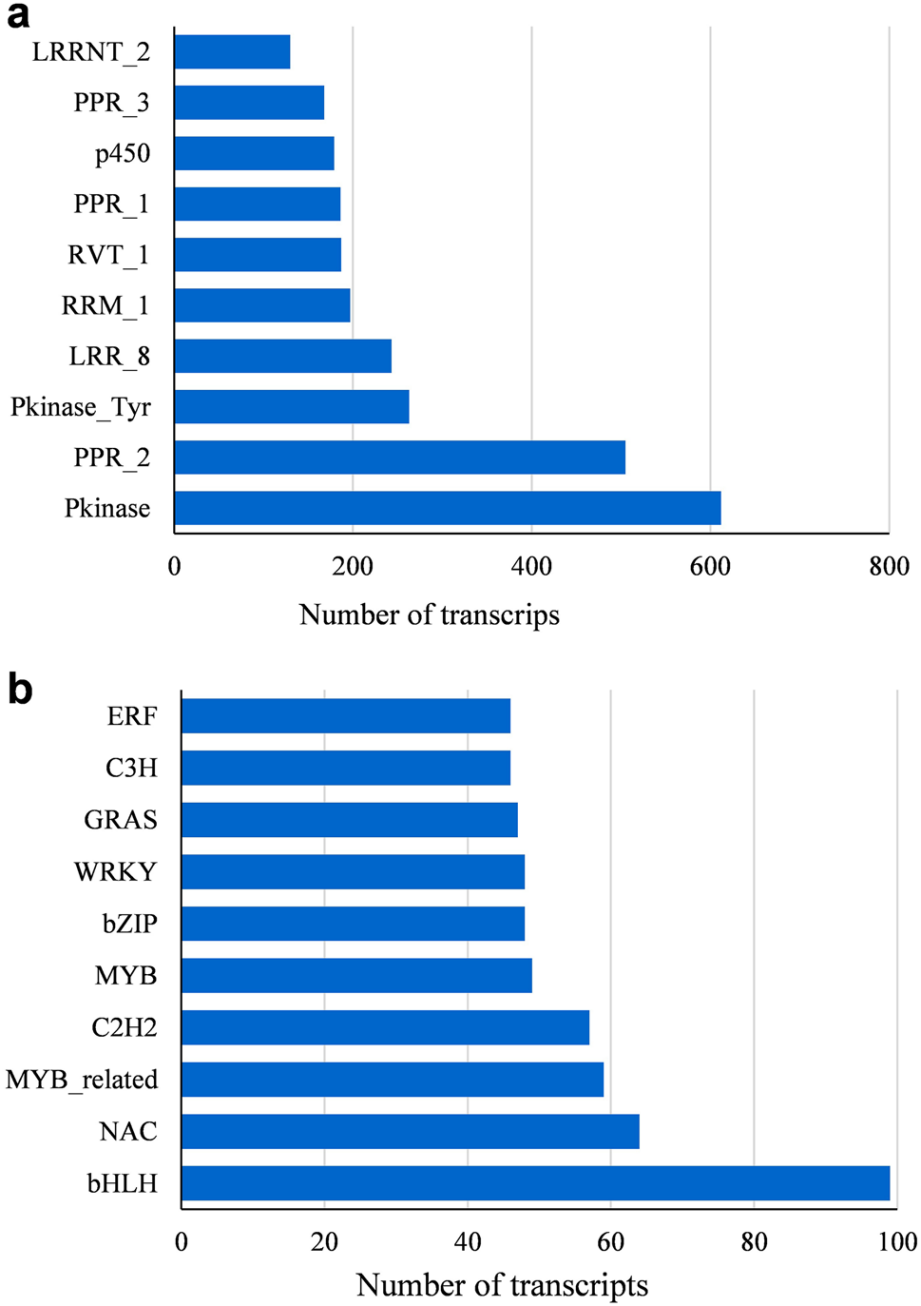
(**a**) The 10 most abundant PFAM protein families in the *I. atropurpurea* a transcriptome. (**b**) The 10 most abundant transcription factors families in the *I. atropurpurea* transcriptome.

In the PFAM analysis, we found 17,385 (9.43%) *Iris* transcripts that contain at least one PFAM protein domain, and that were classified into 3399 Pfam domains/families (supplementary table 1, supplementary info file). The 10 most abundant protein families in *I. atropurpurea* are Pkinase, PPR_2, Pkinase_Tyr, LRR_8, RRM_1, RVT_1, PPR_1, p450, PPR3, and LRRNT_2 (Fig. 6a). Among these protein domains/families, “Protein kinase” and “Tyrosine-protein kinase”, were highly represented. These proteins are known to regulate the activation of most cellular processes^66^, indicating active signal transduction. This is in accordance with our COG results, also showing enrichment of signal transduction genes. Top ranked family is also PPR_2—pentatricopeptide repeats. The PPR family controls varied features of RNA metabolism and plays a profound role in organelle biogenesis and function, e.g. mitochondria and chloroplasts^67–69^. Thus, PPR’s have an essential effect on photosynthesis, respiration, plant development, and environmental responses^69^.

Transcription factors (TFs) are key regulators in biological processes. For prediction of transcription factors, we assigned the protein sequences of all the transcripts to PlantTFDB^54^. We found 1021 transcripts that are predicted to be involved in transcription regulation and were classified into 54 transcription factor families (Fig. 6b, supplementary table 1, supplementary info file). The basic helix–loop–helix (bHLH) transcription factors family was the most abundant in *I. atropurpurea* consisting of 99 gene family members. In plants, the bHLH proteins are associated with a variety of developmental processes, such as trichomes development^70,71^, phytochrome signaling^72^, and cell proliferation and differentiation^70,73^. bHLH proteins have also been shown to interact with other transcription factors such as MYB^71,74^. Furthermore, a protein complex of bHLH and MYB transcription factors, associated with a WD40 repeat protein, regulates various cell differentiation pathways and the anthocyanin biosynthesis pathway^75,76^. The rest of the top 10 TFs are: NAC, MYB-related, C2H2, MYB, bZIP, WRKY, GRAS, C3H and ERF.

In total, we identified 33,033 transcripts in at least one database (supplementary table 1). We were unable to annotate or give a functional prediction to a large fraction of the transcripts. These transcripts could be *Iris* specific genes, genes that have diverged considerably, or genes that are not yet identified in plants.

### Phylogenetic analysis of MADS-box and R2R3-MYB gene families

In the search for orthologous genes involved in flower development in irises, we phylogenetically analyzed two major transcription factor groups, the MADS-box and MYB protein families, to validate the subfamily identities of these genes from *I. atropurpurea*. We performed the phylogenetic analyses using MADS-box and R2R3-MYB protein sequences from *Arabidopsis thaliana* and rice (*Oryza sativa*), the top two annotated species in the transcriptome, and from *Iris fulva*.

#### MADS-box genes

**Figure 7:**
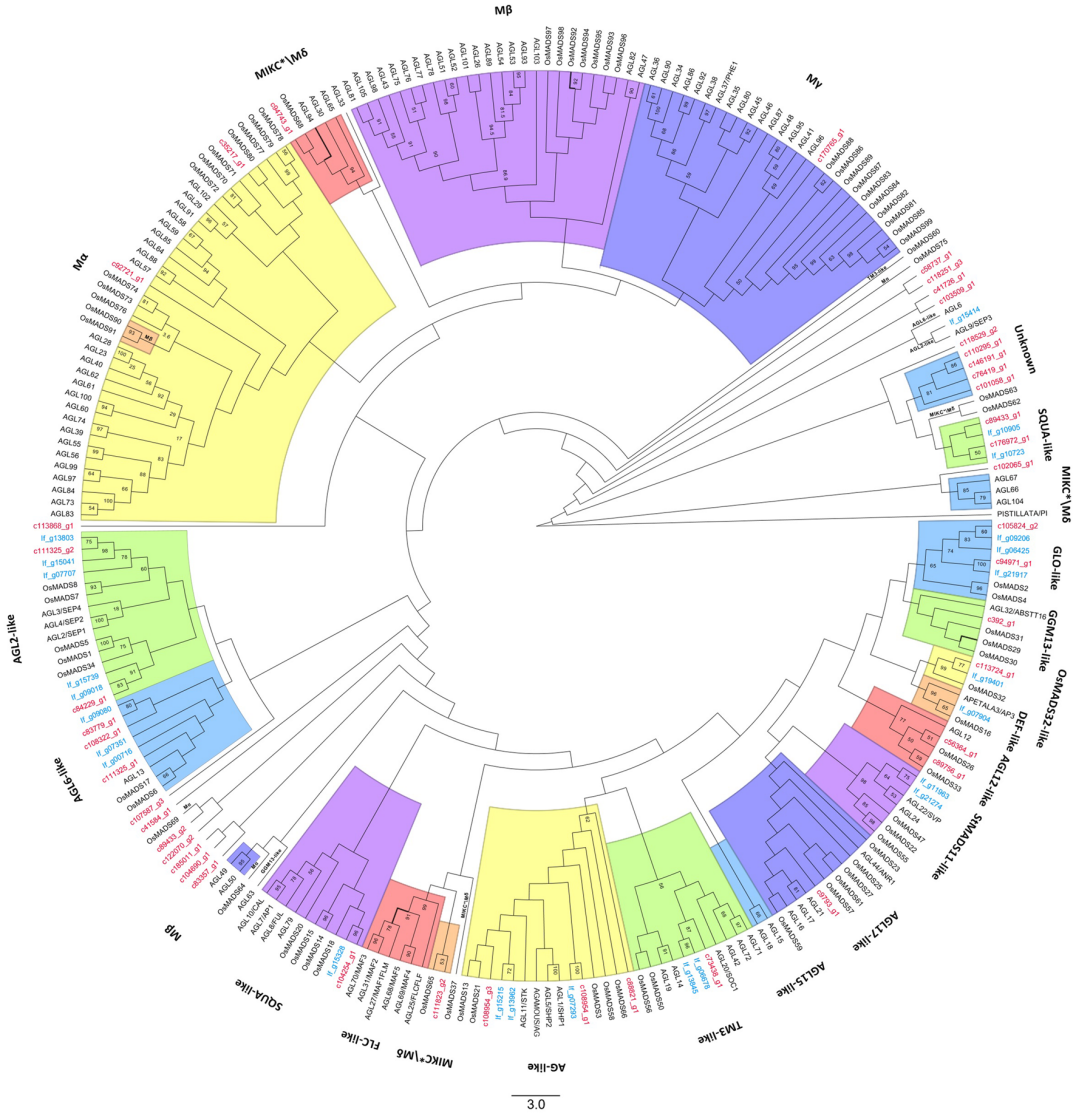
Phylogenetic analysis of MADS-box proteins from the *I. atropurpurea* transcriptome, *I. fulva, Arabidopsis* and rice. *I. atropurpurea* transcripts names are in red and *I. fulva* in light blue. Colours are for visual separation only. Sequences that were separated from their known clade have the name of their original clade written on the branch.

MADS-box proteins, and their complex function, regulate floral organ characteristics and are essential for flower development^28,77,78^. In the *Iris atropurpurea* transcriptome, 43 transcripts were annotated as belonging to the MADS-box family and/or contain the MADS domain. Phylogenetic analysis using *Arabidopsis*, rice, and *I. fulva,* shows orthologous of *I. atropurpurea* in almost all clades of MADS-box proteins (Fig. 7, supplementary table 2). The general organization for most clades was similar to previous comparative phylogenies^19,37^.

Of the *I. atropurpurea* MADS-box genes identified, 19 clustered with MIKCc, 2 with Mα, 0 with Mβ, 1 with Mγ, and 2 grouped with MIKC*/ Mδ-type genes. Among the genes clustered with type II MIKCc MADS, we identified all 14 documented clades^19,37,38^), comprising representative genes of *Arabidopsis*, rice, and *I. fulva*. *I. atropurpurea* had representative transcripts in 10 of the MIKC_C_ clades, except for FLC-like, AGL15-like, DEF-like and StMADS11-like. Similar to previous reports, FLC-like and AGL15-like clades consist only *Arabidopsis* genes, suggesting eudicot specific lineages^19,37,38,79^. Three groups consist *I. atropurpurea* sequences but lack *I. fulva* representatives, AGL12-like, AGL17-like, and GMM13-like. AGL17-like and GMM13-like are not supported by the bootstrap analysis. AGL12-like has three *I. atropurpurea* transcripts, and this clade was well supported. AGL12-like and AGL17-like genes are involved in root development^80,81^, and while the *I. atropurpurea* sequences were derived also from root tissue, the *I. fulva* transcriptome was based on floral and leaves tissues^19^. Four *I. atropurpurea* sequences were clustered alone in a well supported group (81%, designated “Unknown”). These sequences might be of genes unique to *I. atropurpurea.*

Within most of the clades *I. atropurpurea*, *I. fulva* and rice grouped together and *Arabidopsis* sequences grouped together, suggesting a strong species and monocot/eudicot homology. In Arora et.al. *Arabidopsis* and rice also cluster together within the type I MADS clades^37^. Furthermore, a phylogeny of representative type I and II MADS-box genes from several distantly related plant species also showed similar monocot/eudicot separation within clades^33^.

#### R2R3-MYB genes

We found 256 transcripts in the *I. atropurpurea* transcriptome that were annotated as belonging to the MYB family by either trinity or PFAM. Sixty-seven of them were found to have the R2R3-MYB domain. The rest of the transcripts most likely belong to other MYB groups such as R1-MYB, MYB-like proteins, etc., and some might also be incomplete sequences.

**Figure 8:**
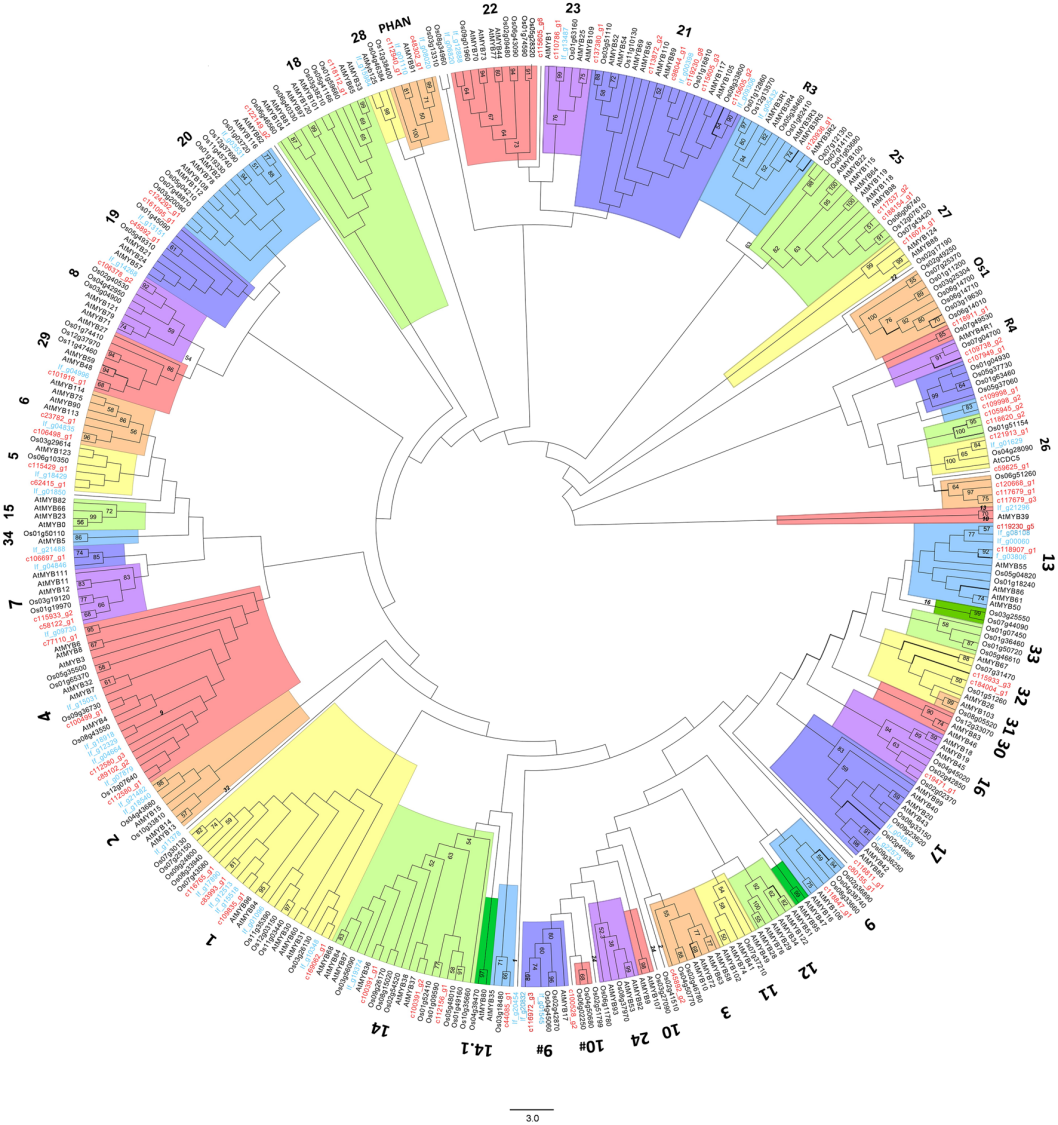
Phylogenetic analysis of R2R3-MYB proteins from the *Iris* transcriptome (highlighted in red), *I. fulva (If), Arabidopsis,* encoded by *AtMYB*, and rice (Oryza sativa, Os). *I. atropurpurea* transcripts names are in red. Colours are for visual separation only.

To create the phylogenetic tree, we aligned the transcripts against R2R3-MYB sequences from *Arabidopsis,* rice, and *I. Fulva* (Fig. 8, supplementary table 3). R3-MYB (R1R2R3) is another major MYB type, that was either the origin of R2R3-MYBs in plants^82^ or evolved from R2R3-MYB^83^, and was also included in the phylogenetic analysis. To analyze the tree, we mainly followed the classification made by Ballerini et al., which consist *Iris* sequences^19^. The organization of the clades in the dendrogram corresponds with that in Ballerini et al., with 26 of the groups supported by bootstrap (> 50%). Several groups showed differences from the phylogeny in Ballerini et al., mostly in the form of a sequence clustered to a different clade, and in most cases not supported by bootstrap. Some major differences were observed for example in group 10, which was separated into 2 clades in our analysis, one with the *Arabidopsis* sequences and one with rice. Similar separation was found in Du et.al., in which the rice sequences are in a separated clade with *Zea Maize*, designated as S42^84^. In our tree, group 16 was also separated into 2 clades, in accordance with other published MYB trees^42,84^.

Fourteen groups of R2R3-MYB genes in the phylogenetic tree lack *I. atropurpurea* representatives, whereas in nine of them *I. fulva* representatives were also lacking, suggesting gene lineages that might not exist in Irises (11, 12, 15, 24, 30, 31, 33, 34, and Os1). Consistent with previous phylogenetic studies, groups 12 and 15 also lack rice representatives, suggesting eudicot specific lineages^19,42^. A comparative analysis of R2R3-MYBs from 50 major eukaryotic lineages showed that group 12 consists only of *Arabidopsis* sequences and that group 15 consists only of eudicot species^84^. Genes in these groups have been shown to control trichome initiation in shoots, root hair patterning, and Cruciferae-specific glucosinolate biosynthesis^41,43,85^. Two of the groups lacking representatives from *Iris*, 33 and Os1, consist only rice genes. Genes from group 33 were previously designated in a monocot-specific clade together with corn (*Zea maize*) sequences^84^. Several groups had only *I. atropurpurea* representatives, lacking *I. fulva,* and vice versa. In addition, in contrast with our expectations, only in a few of groups *I. atropurpurea* and *I. fulva* clustered together within the clade. These observations further support the phylogenetic distance between the two species.

We found two new (bootstrap supported) subgroups consisting only rice and *I. atropurpurea* sequences. Previous phylogenetic studies in other plant species also identified new R2R3-MYB subgroups with no *A. thaliana* representatives. These subgroups might represent genes with specialized functions which were either lost in *Arabidopsis* or obtained after the divergence from the last common ancestor^19,86^. Several *I. atropurpurea* sequences did not cluster together with R2R3-MYBs from any other species, including *I. fulva*. This suggests that these MYB genes might have been acquired in *I. atropurpurea* after divergence within the *Iris* group.

Other MYB and MADS gene groups, which were not identified in our transcriptome, could be genes that were not conserved in irises. Alternatively, these genes might be expressed in earlier stages of flowering initiation, before the appearance of buds^45^, and thus undetected in the transcriptome. In *Iris lortetii*, it was shown that flower organs genes are mostly expressed in an early stage, about two months before stem elongation, when the flower meristem is hidden in the rhizome^45^. Possibly this is the stage when more flower development genes can be found; however, this stage was not sampled in this study and will be explored in further research.

### Development and characterization of cDNA-derived SSR markers

**Figure 9:**
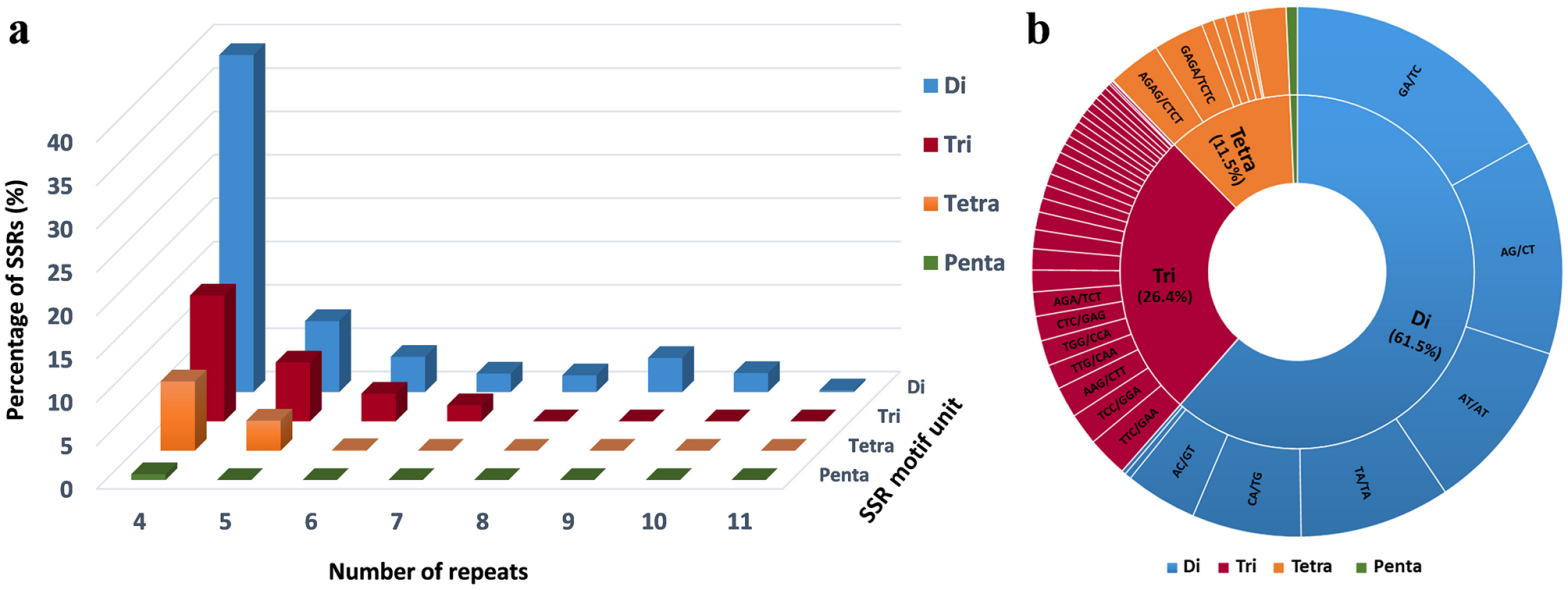
Characterization of SSRs loci found in *Iris* transcriptome. (**a**) Distribution of SSR motif repeat numbers and relative frequency. (**b**) Frequency distribution of SSRs based on motif sequence types.

For the development of new molecular markers, we used all of the 258,466 contigs, generated in this study, to mine potential microsatellites. We defined microsatellites as di- to hexanucleotide SSR with a minimum of four repetitions for all motifs. We identified 1,503 potential SSRs in 1,241 contigs, of which 263 sequences contained more than one SSR. Only 164 of the contigs containing SSRs had annotation and were annotated to 115 genes. We assessed the frequency, type, and distribution of the potential SSRs (Fig. 9). The SSRs included 924 (61.5%) di-nucleotide motifs, 396 (26.4%) tri-nucleotide motifs, 173 (11.5%) tetra-nucleotide motifs, 10 (0.7%) penta-nucleotide motifs, and zero (0%) hexa-nucleotide motifs. The di-, tri-, tetra- and penta-nucleotide repeats had 8, 30, 37 and 9 types of motifs, respectively. The most abundant di-nucleotide type was GA/TC (254, 16.9%), followed by AG/CT (197, 13.1%) and AT/AT (159, 10.6%). The most abundant tri-nucleotide repeat type was TTC/GAA (37, 2.5%).

Di-nucleotide SSRs are usually more common in genomic sequences, whereas tri-nucleotide SSRs are more common in RNA sequences^87–90^. Also, tri-nucleotide repeats are more abundant than dinucleotide repeats in plants^87^. However, in our SSRs, the di-nucleotide repeat type was the most abundant motif detected of all repeat lengths. A higher number of di-nucleotide repeats in RNA sequences has been reported in Louisiana irises^91^, and in other plants such as rubber trees^92^ and *Cajanus cajan* (pigeonpea)^93^. The most abundant di- and tri-nucleotide motifs in *I. atropurpurea* were GA/TC and TTC/GAA, respectively. These results were also coincident with SSRs developed for Louisiana irises, in which the most abundant di- and tri-nucleotide motifs are AG/CT and AAG/CTT^91^.

Until now, SSRs in irises were reported only for Louisiana and Japanese irises^91,94^; however, these SSRs were not transferable to *Oncocyclus* irises (Y. Sapir, un-published). The relatively large set of SSRs obtained from the *I. atropurpurea* transcriptome may enable development of markers for population genetic studies in the Royal Irises.

## Conclusions

In this study, we reported a comprehensive characterization of the transcriptome of *Iris atropurpurea*, an important emerging model for understanding evolutionary processes^3,10–17^. Although transcriptome based on a single replication cannot enable gene expression analysis and extensive biological conclusions, the *Iris* transcriptome established in this study provides a useful database that will increase the molecular resources for the Royal Irises. These resources are currently available only for other iris species^91,94^, which despite belonging to the same genus, they are quite distant from the Royal Irises, hence not easily transferable. In the past decade, many studies have been using transcriptome de novo sequencing and assembly to generate a fundamental source of data for biological research^19,20,95–97^. We generated a substantial number of transcript sequences that can be used for the discovery of novel genes, and specifically genes involved in flower development in irises.

While we did not perform a complete analysis of MADS and R2R3 MYB evolution, we mainly aimed to identify flower development genes and classify their function, and thus provide a framework for the *Iris* genes sequenced in this study. The numerous SSR markers identified will enable the construction of genetic maps and answering important questions in population genetics and conservation. Although genetic studies are still in their early stages in the Royal Irises, we believe that our transcriptome will significantly support and encourage future evolutionary-genetic research in this ecologically important group.

## Supplementary Information

Supplementary Information 1.

Supplementary Information 2.

## Data Availability

Sequences generated in this study were deposited in NCBI’s Gene Expression Omnibus (GEO, http://www.ncbi.nlm.nih.gov/geo/) under the GEO accession number GSE121786.

## References

[1] Matthews V (1997). A guide to species irises: Their identification and cultivation. Edinb. J. Bot..

[2] Makarevitch I, Golovnina K, Scherbik S, Blinov A (2003). Phylogenetic relationships of the siberian *Iris* species inferred from noncoding chloroplast DNA sequences. Int. J. Plant Sci..

[3] Wilson CA, Padiernos J, Sapir Y (2016). The royal irises (*Iris* subg. Iris sect. *Oncocyclus*): Plastid and low-copy nuclear data contribute to an understanding of their phylogenetic relationships. Taxon.

[4] Shmida A, Pollak G (2007). Red Data Book: Endangered Plants of Israel.

[5] Sapir Y, Shmida A (2002). Species concepts and ecogeographical divergence of *Oncocyclus* irises. Israel J. Plant Sci..

[6] Avishai M, Zohary D (1977). Chromosomes in the *Oncocyclus* Irises. Bot. Gaz..

[7] Rice A (2015). The chromosome counts database (CCDB)—a community resource of plant chromosome numbers. New Phytol..

[8] Kentner EK, Arnold ML, Wessler SR (2003). Characterization of high-copy-number retrotransposons from the large genomes of the louisiana *iris* species and their use as molecular markers. Genetics.

[9] Avishai M, Zohary D (1980). Genetic affinities among the *Oncocyclus* irises. Botan. Gaztte.

[10] Arafeh RM (2002). Patterns of genetic and phenotypic variation in Iris haynei and *I. atrofusca* (Iris sect. Oncocyclus the royal irises) along an ecogeographical gradient in Israel and the West Bank. Mol. Ecol..

[11] Dorman M, Sapir Y, Volis S (2009). Local adaptation in four *Iris* species tested in a common-garden experiment. Biol. J. Lin. Soc..

[12] Lavi R, Sapir Y (2015). Are pollinators the agents of selection for the extreme large size and dark color in *Oncocyclus* irises?. New Phytol..

[13] Sapir Y, Mazzucco R (2012). Post-zygotic reproductive isolation among populations of *Iris atropurpurea*: the effect of spatial distance among crosses and the role of inbreeding and outbreeding depression in determining niche width. Evol. Ecol. Res..

[14] Sapir Y, Shmida A, Ne'eman G (2005). Pollination of *Oncocyclus* irises (*Iris*: Iridaceae) by night-sheltering male bees. Plant Biol..

[15] Sapir Y, Shmida A, Ne'eman G (2006). Morning floral heat as a reward to the pollinators of the *Oncocyclus* irises. Oecologia.

[16] Volis S, Blecher M, Sapir Y (2010). Application of complex conservation strategy to *Iris atrofusca* of the Northern Negev, Israel. Biodivers. Conserv..

[17] Yardeni G, Tessler N, Imbert E, Sapir Y (2016). Reproductive isolation between populations of *Iris atropurpurea* is associated with ecological differentiation. Ann. Botany.

[18] Jain M (2011). A next-generation approach to the characterization of a non-model plant transcriptome. Curr. Sci..

[19] Ballerini ES, Mockaitis K, Arnold ML (2013). Transcriptome sequencing and phylogenetic analysis of floral and leaf MIKC(C) MADS-box and R2R3 MYB transcription factors from the monocot *Iris fulva*. Gene.

[20] Tian S (2015). Transcriptome profiling of louisiana iris root and identification of genes involved in lead-stress response. Int. J. Mol. Sci..

[21] Gu C-S (2017). De novo characterization of the Iris lactea var. chinensis transcriptome and an analysis of genes under cadmium or lead exposure. Ecotoxicol. Environ. Saf..

[22] Gu C (2018). De novo sequencing, assembly, and analysis of Iris lactea var. chinensis roots’ transcriptome in response to salt stress. Plant Physiol. Biochem..

[23] Wilson CA (2011). Subgeneric classification in *Iris* re-examined using chloroplast sequence data. Taxon.

[24] Wilson CA (2014). The complete plastid genome sequence of *Iris gatesii* (section *Oncocyclus*), a bearded species from southeastern Turkey. Aliso.

[25] Sapir Y (2016). Iris atropurpurea.

[26] Sapir Y, Shmida A, Fragman O (2003). Constructing red numbers for setting conservation priorities of endangered plant species: Israeli flora as a test case. J. Nat. Conserv..

[27] Watts S, Sapir Y, Segal B, Dafni A (2013). The endangered *Iris atropurpurea* (Iridaceae) in Israel: Honey-bees, night-sheltering male bees and female solitary bees as pollinators. Ann. Bot..

[28] Heijmans K, Morel P, Vandenbussche M (2012). MADS-box genes and floral development: The dark side. J. Exp. Bot..

[29] Glover B (2014). Understanding Flowers and Flowering.

[30] Par̆enicová, L. (2003). Molecular and phylogenetic analyses of the complete MADS-box transcription factor family in Arabidopsis. New Openings MADS World.

[31] De Bodt S (2003). Genomewide structural annotation and evolutionary analysis of the type I MADS-box genes in plants. J. Mol. Evol..

[32] Gramzow L, Ritz MS, Theißen G (2010). On the origin of MADS-domain transcription factors. Trends Genet..

[33] Gramzow L, Theissen G (2010). A hitchhiker's guide to the MADS world of plants. Genome Biol..

[34] Henschel K (2002). Two ancient classes of MIKC-type MADS-box genes are present in the moss Physcomitrella patens. Mol. Biol. Evol..

[35] Schwarz-Sommer Z, Huijser P, Nacken W, Saedler H, Sommer H (1990). Genetic control of flower development by homeotic genes in *Antirrhinum majus*. Science.

[36] Coen ES, Meyerowitz EM (1991). The war of the whorls: Genetic interactions controlling flower development. Nature.

[37] Arora R (2007). MADS-box gene family in rice: Genome-wide identification, organization and expression profiling during reproductive development and stress. BMC Genom..

[38] Becker A, Theißen G (2003). The major clades of MADS-box genes and their role in the development and evolution of flowering plants. Mol. Phylogenet. Evol..

[39] De Bodt S, Raes J, Van de Peer Y, Theißen G (2003). And then there were many: MADS goes genomic. Trends Plant Sci..

[40] Stracke R, Werber M, Weisshaar B (2001). The R2R3-MYB gene family in Arabidopsis thaliana. Curr. Opin. Plant Biol..

[41] Dubos C (2010). MYB transcription factors in Arabidopsis. Trends Plant Sci..

[42] Yanhui C (2006). The MYB transcription factor superfamily of arabidopsis: Expression analysis and phylogenetic comparison with the rice MYB family. Plant Mol. Biol..

[43] Ambawat S, Sharma P, Yadav NR, Yadav RC (2013). MYB transcription factor genes as regulators for plant responses: An overview. Physiol. Mol. Biol. Plants.

[44] Du H (2009). Biochemical and molecular characterization of plant MYB transcription factor family. Biochem. Mosc..

[45] Perl A (1984). The control of flowering and the in vitro propagation of Iris lortetii M. Sc. thesis.

[46] Grabherr MG (2011). Full-length transcriptome assembly from RNA-Seq data without a reference genome. Nat. Biotechnol..

[47] Gurevich A, Saveliev V, Vyahhi N, Tesler G (2013). QUAST: Quality assessment tool for genome assemblies. Bioinformatics.

[48] Simão FA, Waterhouse RM, Ioannidis P, Kriventseva EV, Zdobnov EM (2015). BUSCO: Assessing genome assembly and annotation completeness with single-copy orthologs. Bioinformatics.

[49] Li B, Dewey CN (2011). RSEM: accurate transcript quantification from RNA-Seq data with or without a reference genome. BMC Bioinform..

[50] El-Gebali S (2018). The Pfam protein families database in 2019. Nucleic Acids Res..

[51] Altschul SF, Gish W, Miller W, Myers EW, Lipman DJ (1990). Basic local alignment search tool. J. Mol. Biol..

[52] Huerta-Cepas J (2017). Fast genome-wide functional annotation through orthology assignment by eggNOG-mapper. Mol. Biol. Evol..

[53] Huerta-Cepas J (2019). eggNOG 5.0: A hierarchical, functionally and phylogenetically annotated orthology resource based on 5090 organisms and 2502 viruses. Nucleic Acids Res..

[54] Tian F, Yang D-C, Meng Y-Q, Jin J, Gao G (2019). PlantRegMap: Charting functional regulatory maps in plants. Nucleic Acids Res..

[55] Wernersson R (2006). Virtual Ribosome—a comprehensive DNA translation tool with support for integration of sequence feature annotation. Nucleic Acids Res..

[56] Capella-Gutiérrez S, Silla-Martínez JM, Gabaldón T (2009). trimAl: A tool for automated alignment trimming in large-scale phylogenetic analyses. Bioinformatics (Oxford, England).

[57] Thompson JD, Higgins DG, Gibson TJ (1994). CLUSTAL W: Improving the sensitivity of progressive multiple sequence alignment through sequence weighting, position-specific gap penalties and weight matrix choice. Nucleic Acids Res..

[58] Kumar S, Stecher G, Li M, Knyaz C, Tamura K (2018). MEGA X: Molecular evolutionary genetics analysis across computing platforms. Mol. Biol. Evol..

[59] Jones DT, Taylor WR, Thornton JM (1992). The rapid generation of mutation data matrices from protein sequences. Bioinformatics.

[60] Rambaut, A. FigTree, a graphical viewer of phylogenetic trees. (2007).

[61] Barker MS (2010). EvoPipes. net: Bioinformatic tools for ecological and evolutionary genomics. Evol. Bioinform..

[62] Bou Dagher-Kharrat M (2013). Nuclear DNA C-values for biodiversity screening: Case of the Lebanese flora. Plant Biosyst..

[63] Samad NA (2020). Genome size evolution and dynamics in iris, with special focus on the section oncocyclus. Plants.

[64] Mudalkar S, Golla R, Ghatty S, De Reddy AR (2014). novo transcriptome analysis of an imminent biofuel crop, *Camelina sativa* L. using Illumina GAIIX sequencing platform and identification of SSR markers. Plant Mol. Biol..

[65] Yu C, Xu S, Yin Y (2016). Transcriptome analysis of the Taxodium ‘Zhongshanshan 405’roots in response to salinity stress. Plant Physiol. Biochem..

[66] Lehti-Shiu MD, Shiu S-H (2012). Diversity, classification and function of the plant protein kinase superfamily. Philos. Trans. R. Soc. B Biol. Sci..

[67] Filipovska A, Rackham O (2013). Pentatricopeptide repeats. RNA Biol..

[68] Lurin C (2004). Genome-wide analysis of Arabidopsis pentatricopeptide repeat proteins reveals their essential role in organelle biogenesis. Plant Cell.

[69] Barkan A, Small I (2014). Pentatricopeptide repeat proteins in plants. Annu. Rev. Plant Biol..

[70] Morohashi K (2007). Participation of the Arabidopsis bHLH factor GL3 in trichome initiation regulatory events. Plant Physiol..

[71] Zhao M, Morohashi K, Hatlestad G, Grotewold E, Lloyd A (2008). The TTG1-bHLH-MYB complex controls trichome cell fate and patterning through direct targeting of regulatory loci. Development.

[72] Duek PD, Fankhauser C (2005). bHLH class transcription factors take centre stage in phytochrome signalling. Trends Plant Sci..

[73] Vera-Sirera F (2015). A bHLH-based feedback loop restricts vascular cell proliferation in plants. Dev. Cell.

[74] Zimmermann IM, Heim MA, Weisshaar B, Uhrig JF (2004). Comprehensive identification of *Arabidopsis thaliana* MYB transcription factors interacting with R/B-like BHLH proteins. Plant J..

[75] Goff SA, Cone KC, Chandler VL (1992). Functional analysis of the transcriptional activator encoded by the maize B gene: Evidence for a direct functional interaction between two classes of regulatory proteins. Genes Dev..

[76] Ramsay NA, Glover BJ (2005). MYB–bHLH–WD40 protein complex and the evolution of cellular diversity. Trends Plant Sci..

[77] Honma T, Goto K (2001). Complexes of MADS-box proteins are sufficient to convert leaves into floral organs. Nature.

[78] Theißen G, Saedler H (2001). Floral quartets. Nature.

[79] Zhao T (2006). Characterization and expression of 42 MADS-box genes in wheat (*Triticum aestivum* L.). Mol. Genet. Genom..

[80] Tapia-López R (2008). An AGAMOUS-related MADS-box gene, XAL1 (AGL12), regulates root meristem cell proliferation and flowering transition in Arabidopsis. Plant Physiol..

[81] Zhang H, Forde BG (1998). An Arabidopsis MADS box gene that controls nutrient-induced changes in root architecture. Science.

[82] Rosinski JA, Atchley WR (1998). Molecular evolution of the Myb family of transcription factors: Evidence for polyphyletic origin. J. Mol. Evol..

[83] Jiang C, Gu J, Chopra S, Gu X, Peterson T (2004). Ordered origin of the typical two- and three-repeat Myb genes. Gene.

[84] Du H (2015). The evolutionary history of R2R3-MYB proteins across 50 eukaryotes: New insights into subfamily classification and expansion. Sci. Rep..

[85] Li Y (2013). Novel insights into the function of Arabidopsis R2R3-MYB transcription factors regulating aliphatic glucosinolate biosynthesis. Plant Cell Physiol..

[86] Wilkins O, Nahal H, Foong J, Provart NJ, Campbell MM (2009). Expansion and diversification of the <em>Populus</em> R2R3-MYB family of transcription factors. Plant Physiol..

[87] Varshney RK, Graner A, Sorrells ME (2005). Genic microsatellite markers in plants: Features and applications. Trends Biotechnol..

[88] Thiel T, Michalek W, Varshney R, Graner A (2003). Exploiting EST databases for the development and characterization of gene-derived SSR-markers in barley (*Hordeum vulgare* L.). Theor. Appl. Genet..

[89] Varshney RK, Thiel T, Stein N, Langridge P, Graner A (2002). In silico analysis on frequency and distribution of microsatellites in ESTs of some cereal species. Cell. Mol. Biol. Lett..

[90] Luo M (2005). Generation of expressed sequence tags (ESTs) for gene discovery and marker development in cultivated peanut. Crop Sci..

[91] Tang S (2009). EST and EST-SSR marker resources for *Iris*. BMC Plant Biol..

[92] Li D, Deng Z, Qin B, Liu X, Men ZD (2012). novo assembly and characterization of bark transcriptome using Illumina sequencing and development of EST-SSR markers in rubber tree (Hevea brasiliensis Muell. Arg.). BMC Genom..

[93] Raju NL (2010). The first set of EST resource for gene discovery and marker development in pigeonpea (*Cajanus cajan* L). BMC Plant Biol..

[94] Sun MZ (2012). Genomic and EST-derived microsatellite markers for *Iris laevigata* (Iridaceae) and other congeneric species. Am. J. Bot..

[95] Meyer E (2009). Sequencing and de novo analysis of a coral larval transcriptome using 454 GSFlx. BMC Genom..

[96] Zhang J (2012). De novo assembly and characterisation of the transcriptome during seed development, and generation of genic-SSR markers in Peanut (*Arachis hypogaea* L.). BMC Genom..

[97] Kamenetsky R (2015). Integrated transcriptome catalogue and organ-specific profiling of gene expression in fertile garlic (*Allium sativum* L.). BMC Genom..

