# Author Correction: De novo transcriptome characterization of *Iris atropurpurea* (the Royal Iris) and phylogenetic analysis of MADS-box and R2R3-MYB gene families

**DOI:** 10.1038/s41598-021-97920-1

**Published:** 2021-09-07

**Authors:** Yamit Bar-Lev, Esther Senden, Metsada Pasmanik-Chor, Yuval Sapir

**Affiliations:** 1 The Botanical Garden, School of Plant Sciences and Food Security, G.S. Wise Faculty of Life Science, Tel Aviv University, Tel Aviv, Israel; 2 Bioinformatics Unit, G.S. Wise Faculty of Life Science, Tel Aviv University, 69978 Tel Aviv, Israel

Correction to: *Scientific Reports* https://doi.org/10.1038/s41598-021-95085-5, published online 10 August 2021

The original version of this Article contained errors in the spelling of the authors Yamit Bar-Lev, Esther Senden, Metsada Pasmanik-Chor & Yuval Sapir which were incorrectly given as Bar-Lev Yamit, Senden Esther, Pasmanik-Chor Metsada & Sapir Yuval.

The original Article and accompanying Supplementary Information file have been corrected.

