# Supplementary material for: De novo transcriptome characterization of Iris atropurpurea (the Royal Iris) and phylogenetic analysis of MADS-box and R2R3-MYB gene families
Source: Sci Rep. 2021 Aug 10;11:16246. doi: 10.1038/s41598-021-95085-5 (PMC8355218; doi:10.1038/s41598-021-95085-5)
Supplement: Supplementary file 2 — Supplementary Information 2. [file 41598_2021_95085_MOESM2_ESM.pdf]

De novo Transcriptome Characterization of *Iris atropurpurea* (the Royal Iris) and Phylogenetic Analysis of MADS-box and R2R3-MYB Gene Families

Yamit Bar-Lev [1*], Esther Senden [1], Metsada Pasmanik-Chor [2], and Yuval Sapir [1]

[1] The Botanical Garden, School of Plant Sciences and Food Security, G.S. Wise Faculty of Life Science, Tel Aviv University, Israel

[2] Bioinformatics Unit, G.S. Wise Faculty of Life Science, Tel Aviv University, Tel Aviv 69978, Israel

Yamit Bar-Lev abargily@tauex.tau.ac.il, 972-6407354, Author for correspondence

ORCID id: https://orcid.org/0000-0002-2850-859X

Esther Senden esthersenden@gmail.com

Metsada Pasmanik-Chor metsada@post.tau.ac.il

Yuval Sapir sapiry@tauex.tau.ac.il

Supplementary table 1. Summary of transcripts annotations.

Data base	No. of transcripts
UniProt	28,708
GO	12,623
COG	22,564
PFAM	17,385
TF	1,021
Total	33,033

Supplementary table 2. Phylogenetic analysis of MADS-box genes

		**Arabidopsis**		**Rice**		***Iris fulva***		***Iris atropurpurea***
Group	**Support**	**Name**	**LOC ID**	**Name**	**LOC ID**	**Isogroup**	**GenBank**	**Transcript name**
AG2-like	0.3	AGL2/SEP1	At5g15800	OsMADS1	LOC_Os03g11614	If_g07707	JW988048	c111325_g2
		AGL3/SEP4	At2g03710	OsMADS34	LOC_Os03g54170	If_g09018	JW988050	c84229_g1
		AGL4/SEP2	At3g02310	OsMADS5	LOC_Os06g06750	If_g13803	JW988056	
				OsMADS7	LOC_Os08g41950	If_g15041	JW988059	
				OsMADS8	LOC_Os09g32948	If_g15739	JW988063	
AG6-like	0	AGL13	At3g61120	OsMADS17	LOC_Os04g49150	If_g00716	JW988043	c108322_g1
				OsMADS6	LOC_Os02g45770	If_g07351	JW988047	c111325_g1
						If_g09080	JW988051	c83779_g1
AGL12-like	*77	AGL12	At1g71692	OsMADS26	LOC_Os08g02070			c56364_g1
				OsMADS33	LOC_Os12g10520			c89756_g1
AGL15-like	*66	AGL15	At5g13790					
		AGL18	At3g57390					
AGL17-like	18	AGL16	At3g57230	OsMADS23	LOC_Os08g33488			c9793_g1
		AGL17	At2g22630	OsMADS25	LOC_Os04g23910			
		AGL21	At4g37940	OsMADS27	LOC_Os02g36924			
		AGL44/ANR1	At2g14210	OsMADS57	LOC_Os02g49840			
				OsMADS59	LOC_Os06g23950			
				OsMADS61	LOC_Os04g38770			
AG-like	*62	AGAMOUS/AG	At4g18960	OsMADS13	LOC_Os12g10540	If_g07293	JW988046	c108954_g1
		AGL1/SHP1	At3g58780	OsMADS21	LOC_Os01g66290	If_g13962	JW988058	c108954_g3
		AGL11/STK	At4g09960	OsMADS3	LOC_Os01g10504	If_g15215	JW988060	c88821_g1
		AGL5/SHP2	At2g42830	OsMADS58	LOC_Os05g11414			
				OsMADS66	LOC_Os05g11380			
DEF-like	*96	APETALA3/AP3	At3g54340	OsMADS16	LOC_Os06g49840	If_g07904	JW988049	
FLC-like		AGL25/FLCFLF	At5g10140					

		AGL27/MAF1FLM	At1g77080					
		AGL31/MAF2	At5g65050					
		AGL68/MAF5	At5g65080					
		AGL69/MAF4	At5g65070					
		AGL70/MAF3	At5g65060					
GGM13-like	41	AGL32/ABSTT16	At5g23260	OsMADS29	LOC_Os02g07430			c392_g1
				OsMADS30	LOC_Os06g45650			
				OsMADS31	LOC_Os04g52410			
GLO-like	*65			OsMADS2	LOC_Os01g66030	If_g06425	JW988044	c105824_g2
				OsMADS4	LOC_Os05g34940	If_g09206	JW988052	c94971_g1
						If_g21917	JW988066	
SQUA-like	9.4	AGL10/CAL	At1g26310	OsMADS14	LOC_Os03g54160	If_g10723	JW988053	c104254_g1
		AGL7/AP1	At1g69120	OsMADS15	LOC_Os07g01820			
		AGL79	At3g30260	OsMADS18	LOC_Os07g41370			
		AGL8/FUL	At5g60910	OsMADS20	LOC_Os12g31748			
	35					If_g10905	JW988054	c176972_g1
						If_g15328	JW988061	c89433_g1
StMADS11-like	*98	AGL22/SVP	At2g22540	OsMADS22	LOC_Os02g52340	If_g11963	JW988055	
		AGL24	At4g24540	OsMADS47	LOC_Os03g08754	If_g21274	JW988065	
				OsMADS55	LOC_Os06g11330			
TM3-like	*56	AGL14	At4g11880	OsMADS50	LOC_Os03g03100	If_g06678	JW988045	c73438_g1
		AGL19	At4g22950	OsMADS56	LOC_Os10g39130	If_g13845	JW988057	
		AGL20/SOC1	At2g45660					
		AGL42	At5g62165					
		AGL71	At5g51870					
		AGL72	At5g51860					
OsMADS32-like	*99			OsMADS32	LOC_Os01g52680	If_g19401	JW988064	c113724_g1
M alpha	0	AGL100	At1g17310	OsMADS70	LOC_Os05g23780			c35217_g1

		AGL102	At1g47760	OsMADS71	LOC_Os06g22760		c92721_g1
		AGL23	At1g65360	OsMADS72	LOC_Os03g14850		
		AGL28	At1g01530	OsMADS73	LOC_Os12g21850		
		AGL29	At2g34440	OsMADS74	LOC_Os12g21880		
		AGL39	At5g27130	OsMADS76	LOC_Os06g30830		
		AGL40	At4g36590	OsMADS77	LOC_Os09g02780		
		AGL55	At1g60920	OsMADS78	LOC_Os09g02830		
		AGL56	At1g60880	OsMADS79	LOC_Os01g74440		
		AGL57	At3g04100	OsMADS80	LOC_Os02g06860		
		AGL58	At1g28450				
		AGL59	At1g28460				
		AGL60	At1g72350				
		AGL61	At2g24840				
		AGL62	At5g60440				
		AGL64	At1g29960				
		AGL73	At5g38620				
		AGL74	At1g48150				
		AGL83	At5g49490				
		AGL84	At5g49420				
		AGL85	At1g54760				
		AGL88	At2g11990				
		AGL91	At3g66656				
		AGL97	At1g46408				
		AGL99	At5g04640				
M beta	2.1	AGL101	At5g27050	OsMADS92	LOC_Os01g23750		
		AGL103	At3g18650	OsMADS93	LOC_Os01g23760		
		AGL105	At5g37420	OsMADS94	LOC_Os01g23770		
		AGL26	At5g26870	OsMADS95	LOC_Os01g23780		
		AGL43	At5g40220	OsMADS96	LOC_Os01g67890		

		AGL47	At5g55690	OsMADS97	LOC_Os01g68420		
		AGL51	At4g02235	OsMADS98	LOC_Os01g68560		
		AGL52	At4g11250				
		AGL53	At5g27070				
		AGL54	At5g27090				
		AGL75	At5g41200				
		AGL76	At5g40120				
		AGL77	At5g38740				
		AGL78	At5g65330				
		AGL81	At5g39750				
		AGL82	At5g58890				
		AGL89	At5g27580				
		AGL93	At5g26950				
		AGL98	At5g39810				
	*93			OsMADS90	LOC_Os07g04170		
				OsMADS91	LOC_Os01g11510		
	*95	AGL49	At1g60040				
		AGL50	At1g59810				
M gamma	21.5	AGL34	At5g26580	OsMADS81	LOC_Os04g24790		c170765_g1
		AGL35	At5g26630	OsMADS82	LOC_Os04g24800		
		AGL36	At5g26650	OsMADS83	LOC_Os04g24810		
		AGL37/PHE1	At1g65330	OsMADS84	LOC_Os04g25870		
		AGL38	At1g65300	OsMADS85	LOC_Os04g25920		
		AGL41	At2g26880	OsMADS86	LOC_Os03g37670		
		AGL45	At3g05860	OsMADS87	LOC_Os03g38610		
		AGL46	At2g28700	OsMADS88	LOC_Os01g18420		
		AGL48	At2g40210	OsMADS89	LOC_Os01g18440		
		AGL80	At5g48670	OsMADS99	LOC_Os04g25930		
		AGL86	At1g31630				

		AGL87	At1g22590				
		AGL90	At5g27960				
		AGL92	At1g31640				
		AGL95	At2g15660				
		AGL96	At5g06500				
MIKC*\M delta	13	AGL30	At2g03060	OsMADS68	LOC_Os11g43740		c94743_g1
		AGL65	At1g18750				
		AGL94	At1g69540				
	*85	AGL66	At1g77980				
		AGL67	At1g77950				
		AGL104	At1g22130				
	48			OsMADS62	LOC_Os08g38590		
				OsMADS63	LOC_Os06g11970		
	*53			OsMADS65	LOC_Os01g69850		c111823_g2
Unknown	*81						c101058_g1
							c110295_g1
							c146191_g1
							c76419_g1

*support greater than 50

The MIKC*\M delta sequences are clustered in several groups, some of them between MIKCc clades

Supplementary table 3. Phylogenetic analysis of R2R3-MYB genes

		Arabidopsis		Rice		Iris fulva		Iris atropurpurea
**Group**	**Support**	**Name**	**LOC ID**	**LOC ID**	**Pseudomol**	**Isogroup**	**GenBank**	**Transcript name**
**1**	8.3	AtMYB30	At3g28910	Os03g26130		If_g01096	JW988002	c109835_g1
		AtMYB31	At1g74650	Os07g25150		If_g10348	JW988021	c116765_g1
		AtMYB60	At1g08810	Os07g30130		If_g12513	JW988024	c169062_g1
		AtMYB94	At3g47600	Os07g43580	13107.m04579	If_g15518	JW988030	c83993_g1
		AtMYB96	At5g62470	Os08g33940		If_g17890	JW988032	
				Os09g24800				
				Os11g03440	13111.m00283			
				Os11g35390				
				Os12g03150	13112.m00270			
**2**	0.5	AtMYB13	At1g06180	Os04g43680		If_g18540	JW988034	
		AtMYB14	At2g31180	Os10g33810		If_g21482	JW988040	
		AtMYB15	At3g23250					
**3**	*55	AtMYB10	At3g12820	Os02g46780				c45892_g2
		AtMYB58	At1g16490	Os04g50770	13104.m05157			
		AtMYB63	At1g79180					
		AtMYB72	At1g56160					
**4**	2.1	AtMYB3	At1g22640	Os01g65370		If_g04664	JW988009	c100499_g1
		AtMYB32	At4g34990	Os05g35500		If_g07879	JW988016	c112580_g1
		AtMYB4	At4g38620	Os08g43550		If_g09730	JW988020	c112580_g3
		AtMYB6	At4g09460	Os09g36730	13109.m03625	If_g12329	JW988023	c77110_g1
		AtMYB7	At2g16720	Os12g07640		If_g15031	JW988029	c89102_g2
		AtMYB8	At4g09460			If_g18918	JW988035	
**5**	32	AtMYB123	At5g35550	Os03g29614		If_g18429	JW988033	c115429_g1
				Os06g10350				c62415_g1
**6**	*56	AtMYB113	At1g66370			If_g04835	JW988011	c106498_g1
		AtMYB114	At1g66380					c23782_g1
		AtMYB75	At1g56650					
		AtMYB90	At1g66390					
**7**	*83	AtMYB11	At3g62610	Os01g19970	13101.m02169			c115933_g2
		AtMYB111	At5g49330	Os03g19120	13103.m02286			c58122_g1

		AtMYB12	At2g47460					
	*85					If_g04846	JW988012	c106697_g1
						If_g21488	JW988041	
**8**	*59	AtMYB121	At3g30210	Os02g40530	13102.m04521	If_g14268	JW988028	c106378_g2
		AtMYB71	At3g24310	Os03g04900	13103.m00509			
		AtMYB79	At4g13480	Os04g42950				
**9**	24	AtMYB106	At3g01140	Os02g36890	13102.m04034			c118847_g1
		AtMYB16	At5g15310	Os04g38740	13104.m03728			
				Os08g33660				
**9#**	*80	AtMYB17	At3g61250	Os02g42870		If_g01545	JW988004	c116972_g3
				Os04g45060	13104.m04513	If_g20832	JW988038	
**10**	*98	AtMYB107	At3g02940					
		AtMYB9	At5g16770					
**10#**	*68			Os04g50680	13104.m05148			
				Os06g02250				
**11**	*54	AtMYB102	At4g21440	Os07g37210				
		AtMYB41	At4g28110					
		AtMYB49	At5g54230					
		AtMYB74	At4g05100					
**12**	*92	AtMYB122	At1g74080					
		AtMYB28	At5g61420					
		AtMYB29	At5g07690					
		AtMYB34	At5g60890					
		AtMYB51	At1g18570					
		AtMYB76	At5g07700					
**13**	40	AtMYB50	At1g57560	Os01g18240		If_g00060	JW988000	c118907_g1
		AtMYB55	At4g01680	Os05g04820		If_g03806	JW988008	c119230_g5
		AtMYB61	At1g09540			If_g08108	JW988018	
		AtMYB86	At5g26660					
**14**	*54	AtMYB36	At5g57620	Os01g09590	13101.m01025	If_g19374	JW988036	c100391_g1
		AtMYB37	At5g23000	Os01g49160				c100391_g2
		AtMYB38	At2g36890	Os01g52410				c112156_g1
		AtMYB68	At5g65790	Os02g54520	13102.m06245			
		AtMYB84	At3g49690	Os03g56090	13103.m06113			
		AtMYB87	At4g37780	Os05g48010				

				Os08g15020	13108.m01608			
				Os09g26170				
				Os10g35660				
**14.1**	*97	AtMYB80	At5g56110	Os04g39470	13104.m03828			
	*71	AtMYB35	At3g28470	Os03g18480				c44085_g1
**15**	*72	AtMYB0	At3g27920					
		AtMYB23	At5g40330					
		AtMYB66	At5g14750					
		AtMYB82	At5g52600					
**16**	*94	AtMYB18	At4g25560	Os02g02370	13102.m00172			c19471_g1
		AtMYB19	At5g52260	Os02g42850	13102.m12426			
		AtMYB45	At3g48920	Os04g45020	13104.m04507			
**16#**	*98			Os03g25550				
				Os07g44090				
**17**	*83	AtMYB20	At1g66230	Os02g49986		If_g04833	JW988010	
		AtMYB40	At5g14340	Os08g33150		If_g22573	JW988042	
		AtMYB42	At4g12350	Os09g23620				
		AtMYB43	At5g16600	Os09g36250	13109.m03567			
		AtMYB85	At4g22680					
		AtMYB99	At5g62320					
**18**	39	AtMYB101	At2g32460	Os01g59660	13101.m06342			c118112_g1
		AtMYB104	At2g26950	Os03g38210	13103.m04150			
		AtMYB120	At5g55020	Os05g41166				
		AtMYB33	At5g06100	Os06g40330	13106.m04191			
		AtMYB65	At3g11440	Os06g46560	13106.m04920			
		AtMYB81	At2g26960					
		AtMYB97	At4g26930					
**19**	3.8	AtMYB21	At3g27810	Os01g45090		If_g13151	JW988026	c161095_g1
		AtMYB24	At5g40350	Os05g49310				c45892_g1
		AtMYB57	At3g01530					
**20**	11	AtMYB108	At3g06490	Os01g03720		If_g03531	JW988007	c124292_g1
		AtMYB112	At1g48000	Os01g19330	13101.m02102			
		AtMYB116	At1g25340	Os03g20090				
		AtMYB2	At2g47190	Os05g04210				
		AtMYB62	At1g68320	Os07g48870				

		AtMYB78	At5g49620	Os11g45740				
				Os12g37690				
**21**	26	AtMYB105	At1g69560	Os01g16810		If_g00398	JW988001	c113872_g2
		AtMYB110	At3g29020	Os03g51110		If_g09306	JW988019	c115605_g2
		AtMYB117	At1g26780	Os08g33800	13108.m03527			c115605_g3
		AtMYB52	At1g17950	Os11g10130				c119230_g8
		AtMYB54	At1g73410					c137380_g1
		AtMYB56	At5g17800					c98044_g1
		AtMYB69	At4g33450					
		AtMYB89	At5g39700					
**22**	*73	AtMYB44	At5g67300	Os01g74590		If_g06820	JW988015	
		AtMYB70	At2g23290	Os02g09480	13102.m01040	If_g12888	JW988025	
		AtMYB73	At4g37260	Os05g28320				
		AtMYB77	At3g50060	Os06g43090				
				Os09g01960				
**23**	*76	AtMYB1	At3g09230	Os01g63160		If_g13487	JW988027	c110786_g1
		AtMYB109	At3g55730					
		AtMYB25	At2g39880					
**24**	*52	AtMYB53	At5g65230	Os06g11780				
		AtMYB92	At5g10280	Os08g37970				
		AtMYB93	At1g34670					
**25**	*82	AtMYB100	At2g25230	Os01g63680	13101.m06832			c117537_g2
		AtMYB115	At5g40360	Os06g06740	13106.m00696			c188154_g1
		AtMYB118	At3g27780	Os07g12130	13107.m01247			
		AtMYB119	At5g58850	Os07g14110				
		AtMYB22	At5g40430	Os12g07610	13112.m00798			
		AtMYB64	At5g11050					
		AtMYB98	At4g18770					
**26**	*100	AtCDC5	At1g09770	Os04g28090		If_g01629	JW988005	c121913_g1
**27**	*99	AtMYB124	At1g14350	Os07g43420	13107.m04564			c116074_g1
		AtMYB88	At2g02820					
**28**	*98	AtMyb125	At3g60460	Os04g46384	13104.m04667	If_g15984	JW988031	
**29**	*86	AtMYB27	At3g53200	Os01g74410		If_g04996	JW988013	c101916_g1
		AtMYB48	At3g46130	Os11g47460				
		AtMYB59	At5g59780	Os12g37970				

**30**	*90	AtMYB46	At5g12870	Os12g33070				
		AtMYB83	At3g08500					
**31**	*99	AtMYB103	At1g63910	Os08g05520				
**32**	4.3	AtMYB26	At3g13890	Os01g51260	13101.m05343			c115933_g3
		AtMYB67	At3g12720	Os07g31470				c184004_g1
**33**	*58			Os01g07450	13101.m00758			
				Os01g36460	13101.m03692			
				Os01g50720				
				Os05g46610	13105.m04980			
**34**	*86	AtMYB5	At3g13540	Os01g50110				
**Os1**	*100			Os01g11200				
				Os02g49250				
				Os03g19630				
				Os03g25304				
				Os06g14010				
				Os06g14700				
				Os06g14710				
				Os07g25370				
**PHAN**	*100	AtMYB91	At2g37630	Os12g38400		If_g01110	JW988003	c112940_g1
						If_g08020	JW988017	c48302_g1
**R3**	48	AtMYB3R1	At4g32730	Os01g12860	13101.m01405	If_g05432	JW988014	c120936_g1
		AtMYB3R2	At4g00540	Os01g62410	13101.m06678			
		AtMYB3R3	At3g09370	Os05g38460	13105.m04033			
		AtMYB3R4	At5g11510	Os12g13570				
		AtMYB3R5	At5g02320					
**AtMYB4R1**	*91	AtMYB4R1	At3g18100	Os07g04700				c109738_g2
	*99	AtMYB47	At1g18710					
		AtMYB95	At1g74430					
	*85			Os07g49530	13107.m05331			c118911_g1
	*99			Os01g04930	13101.m00460			c107949_g1
				Os01g63460	13101.m06811			
				Os05g37060				
				Os05g37730	13105.m03933			

*83				c109998_g1<br>c109998_g2
*100		Os01g51154 13101.m05331		c105945_g2<br>c118620_g2
*64		Os06g51260 13106.m05530		c117679_g1<br>c117679_g3<br>c120668_g1
*70	AtMYB39 At4g17780		If_g21296 JW988039	
